# Modulation of Peripheral Immune Cell Subpopulations After RapidArc/Moderate Hypofractionated Radiotherapy for Localized Prostate Cancer: Findings and Comparison With 3D Conformal/Conventional Fractionation Treatment

**DOI:** 10.3389/fonc.2022.829812

**Published:** 2022-06-01

**Authors:** Fiorella D’Auria, Teodora Statuto, Luciana Rago, Antonietta Montagna, Giovanni Castaldo, Irene Schirò, Anna Zeccola, Teresa Virgilio, Gabriella Bianchino, Antonio Traficante, Alessandro Sgambato, Vincenzo Fusco, Luciana Valvano, Giovanni Calice

**Affiliations:** ^1^ Laboratory of Clinical Pathology, Centro di Riferimento Oncologico della Basilicata (IRCCS-CROB), Rionero in Vulture, Italy; ^2^ Laboratory of Clinical Research and Advanced Diagnostics, Centro di Riferimento Oncologico della Basilicata (IRCCS-CROB), Rionero in Vulture, Italy; ^3^ Radiotherapy Unit, Centro di Riferimento Oncologico della Basilicata (IRCCS-CROB), Rionero in Vulture, Italy; ^4^ Scientific Direction, Centro di Riferimento Oncologico della Basilicata (IRCCS-CROB), Rionero in Vulture, Italy; ^5^ Laboratory of Preclinical and Translational Research, Centro di Riferimento Oncologico della Basilicata (IRCCS-CROB), Rionero in Vulture, Italy

**Keywords:** prostate cancer, radiotherapy, conventional fractionation, peripheral immune cells, toxicity, hypofractionation

## Abstract

Radiotherapy (RT) is an important therapeutic option in patients with localized prostate cancer (PC). Unfortunately, radiation treatment causes a decrease in peripheral lymphocytes and, consequently, influences the patients’ immune status. Our aim was to study changes in peripheral blood immune cell subpopulations after RT and during 6 months’ follow-up in 2 groups of PC patients irradiated with different techniques and dose fractions with curative intent. We also investigated the presence of correlation between immune cell modulation and genitourinary or gastrointestinal toxicity. We enrolled 44 patients treated with curative RT (RapidArc/hypofractionation regimen or 3D conformal/conventional fractionation) for localized PC. Total white blood cell (WBC), absolute lymphocyte counts (ALCs), and peripheral immune cell subpopulations were analyzed at baseline, at the end of RT, and 3 and 6 months after the end of RT. WBC and ALC greatly decreased at the end of RT with a trend to recover at 6 months’ follow-up in the hypofractionation group but not in the conventional one. Furthermore, B, total T, T CD4+, T CD8+, and NK cell values dropped significantly in both groups at the end of RT, with a minor decrease detectable in the hypofractionation group for B, total T, and T CD4+ lymphocytes with respect to the other technique/fractionation group. Double-negative T (DNT), double-positive T (DPT), and NKT cells significantly decreased at the end of RT with a slight tendency to recover values during follow-up, particularly in the hypofractionation group. No correlation with genitourinary or gastrointestinal toxicity was found. In this study, we showed, for the first time, the effects of RapidArc/moderate hypofractionation RT on immune cell subsets in patients treated for localized PC. Due to the growing interest in minority T-cell subpopulations for immunotherapy, we also reported longitudinal monitoring of the effects of RT on DNT, DPT, and NKT, which was never studied before. Our preliminary data highlight the importance of considering the effects of different RT techniques/fractionation regimens on peripheral immune cells, in the era of RT and immunotherapy combination.

## Introduction

Prostate cancer is the second most frequently diagnosed malignancy in men worldwide (1). Radiotherapy (RT) is an important treatment modality in patients with localized prostate cancer (2). Conventionally, RT is delivered using single doses of 1.8–2.0 Gray (Gy) and a total dose range from 76 to 80 Gy (3). After Brenner and Hall (4) showed that prostatic cancers appear more sensitive to changes in fractionation than most other cancers, as they contain a low proportion of proliferating cells and an α/β value of 1.5, several trials have evaluated the suitability of hypofractionated RT confirming its clinical efficacy and the economic and logistic advantages (5–10).

Unfortunately, during whole-pelvis radiotherapy (WPRT), radiation impairs radiosensitive tissues such as bone marrow (11). The resulting decrease in peripheral leukocyte count, particularly lymphocytes, has been widely reported in literature (12–16). Several authors (17–24) have yet to observe, in different types of cancer, the changes of lymphocyte subpopulations after RT. In particular, Lissoni et al. (17) evidenced that in patients affected by uterine tumors that underwent WPRT, T lymphocyte, CD4+, CD8+, and natural killer (NK) cell mean absolute values significantly decreased but with different behavior. In breast cancer patients, differential effects of RT with and without adjuvant chemotherapy were shown on lymphocyte counts and lymphocyte subpopulation composition (18). Prostate cancer patients treated with carbon ion radiotherapy (CIR) (22) experienced a gradual decrease of CD19+ cells and an increase of CD4+ cells during RT. These variations, together with those of CD3+ and CD8+ counts, can be predictive of outcome for prostate cancer patients after CIR (22). Eckert et al. (23) observed in 18 patients affected by prostate cancer that during the course of standard RT (70–78 Gy) combined (in 83.3% of the cases) with anti-hormonal therapy, percentage of T cells, CD8+ and naïve CD4+ T cells, and B cells decreased while regulatory T and NK cells increased. A further study on a group of 33 prostate cancer patients (24) investigated the different effects of definitive or salvage RT on peripheral lymphocyte subsets. The authors found that they cause similar effects and, in particular, that B lymphocytes are more sensitive to both type of RT with respect to T and NK cells.

Moreover, Yuan and Wang (25) found that T, B, and NK lymphocytes reacted differently to different RT regimens in breast cancer patients.

In addition to the well-known CD4+ T helper and CD8+ T cytotoxic populations, other smaller subgroups of T cells exist in peripheral blood: regulatory T cells (Tregs), a subset of CD4+ T cells with immune suppression functions (26); NKT cells, a subset of T lymphocytes that expresses NK markers (27); and double-negative T (DNT) cells, a subset of T lymphocytes negative for CD4, CD8, and NK cell markers. DNT cells are involved in immune response regulation (28, 29).

The other rare T-cell population present in peripheral blood is CD4+ CD8+ double-positive T (DPT). Its function is controversial, with studies reporting a cytotoxic or suppressive role for these cells (30, 31).

We studied changes of peripheral blood immune cell subpopulations after RT and during 6 months’ follow-up, in a group of PC patients undergoing RapidArc and moderate hypofractionated RT with curative intent. The RT immune effects in these patients were compared with those in a second small group of PC patients treated with 3D conformal/conventional fractionation RT.

## Materials and Methods

### Study Characteristics

Forty-four male patients candidate to curative RT for localized prostate cancer (median age, 76; range, 54–91), who had not received chemotherapy, were prospectively enrolled into our single-institute study that was examined and approved by the local ethics committee (Comitato Etico Unico Regionale per la Basilicata). All the enrolled patients gave their informed consent prior to their inclusion in the study. Thirty-two patients (hypoF group) underwent moderate hypofractionation using volumetric arc intensity modulated radiation therapy technique (RapidArc, RA), with a daily dose/fraction of 3.1 Gy and a total dose to the planned target volume (PTV) of 62 Gy. Twelve patients (standard group) underwent conventional daily fractionation of 2 Gy using a 3D conformal technique (3D-CRT) and a total prescription dose to PTV of 78 Gy (**Table 1**). Patients were selected for the technique in a casual manner, based on the instrumentation availability. During the study, according to our institutional standards, the 3D-CRT has no longer been used for the treatment of this type of patients, in favor of RA. This resulted in the small number of patients in the standard group. Twenty-four (54.5%) patients received hormone therapy, 8 (66.6%) in the standard group and 16 (50%) in the hypoF group.

**Table 1:** Patients’ characteristics.

Characteristics	Observed values (*n* = 44 patients)
**Median age** (range)	76 (54–91)
**Stage**	
T2a	1
T2b	13
T2c	12
T3a	18
**Gleason**	
6 (3 + 3)	13
7 (3 + 4)	13
7 (4 + 3)	5
8 (4 + 4)	13
**Initial PSA** (ng/ml, median, range)	9.73 (0.046–48)
**Risk class**	
Low	15
Intermediate	16
High	13
**Prescription dose to PTV and dose/fraction of 3D conformal RT** (Gy)	78; 2.0
**Prescription dose to PTV and dose/fraction of RA RT** (Gy)	62; 3.1

PTV, planned target volume; RA, RapidArc irradiation technique.

### Treatment Characteristics

The patients were simulated using Combifix (CIVCO, Orange City, IA, USA) and treated by RA RT with Trilogy Linac (Varian Medical System, Palo Alto, CA, USA) and for 3D conformal RT with Clinac 2100 (Varian Medical System, Palo Alto, CA, USA).

The clinical target volume (CTV) was composed of the prostate gland with or without the seminal vesicles. The corresponding PTV was defined as CTV + 8 mm margin in each direction, with the exception of the posterior direction.

### Planning Parameters

The planning object was to cover 95% of each PTV with at least 95% of the prescribed dose and Dmax < 107%. For RA RT, the organ at risk (OAR) planning objectives were as follows: V43.4 Gy < 50%, V51 Gy < 40%, and V59.5 Gy < 25% for the rectum; V51 Gy < 35% for the bladder; Dmean < 45 Gy for the femoral heads; 410 cc < 45 Gy, 740 cc < 30 Gy, and 1,050 cc < 15 Gy for the intestinal cavity. The beam energy used to perform the treatment plans was equal to 6 MV.

For 3D conformal RT, OAR objectives were as follows: V46.5 Gy < 50% and V64.5 Gy < 15% for the rectum; V46.5 Gy < 50% and V64.5 < 20% for the bladder; 410 cc < 45 Gy, 740 cc < 30 Gy, and 1,050 cc < 15 Gy for the intestinal cavity. The beam energy used to perform the treatment plans was equal to 15 MV.

### Flow Cytometry

For flow cytometry analysis, peripheral blood samples were collected before RT (t0), at the end of RT (t1), which is the day when the RT planning was completed, and at follow-up time of 3 (t2) and 6 months (t3) after the end of RT for each patient. At the same time points, absolute counts of white blood cells (WBCs) and lymphocytes (ALC) were determined with a Beckman Coulter DXH800.

The peripheral blood immune cell subpopulations of total T cells (CD3+), T helper cells (CD3+ CD4+), T cytotoxic cells (CD3+ CD8+), regulatory T cells (Tregs) (CD4+ CD25+ CD127low/-), DNT cells (CD3+ CD4- CD8- CD16- CD56-), DPT cells (CD3+ CD4+ CD8+), NKT cells (CD3+ CD16+ CD56+), NK cells (CD3- CD16+ CD56+), and B cells (CD19+) were analyzed by a FACSCanto II flow cytometer (BD Biosciences), as previously reported (32). In brief, fluorescence-labeled antibodies (BD Biosciences) were mixed with 100 µl of peripheral blood and incubated for 15 min in the dark. The antibody combinations were CD3-FITC/CD16CD56-PE/CD45-PerCP/CD8-PE-Cy7/CD4-APC/CD19-APC-Cy7; CD127-PE/CD4-PerCP/CD25-PE-Cy7/CD45-APC-Cy7. For each combination, 50,000 to 100,000 CD45-positive cells were analyzed. Each immune cell subpopulation was indicated as the percentage of the total lymphocyte population, gated using CD45 and SSC data. The absolute number of each cell subpopulation was obtained using the percentage and the ALC values (data summarized in **Table 2**).

**Table 2:** Cell populations’ values at baseline (µl^−1^, median, range).

	RA/hypofractionation RT group (*n* = 32)	3D conformal/conventional fractionation RT group (*n* = 12)
WBC	7,285 (3,340–13,770)	6,835 (5,100–9,700)
ALC	2,150 (800–4,000)	2,100 (1,200–3,300)
B cells	134.6 (25.9–439.4)	137.1 (35.1–260.7)
NK cells	314.5 (104.4–948.5)	506.1 (200–1,120.6)
Total T cells	1,490.8 (575.2–3,104.3)	1,431.1 (868.8–2,039.4)
T CD4+ cells	987 (409.7–1,716.8)	851.8 (542.1–1,531.2)
T CD8+ cells	537.6 (75.2–1,842.6)	419.4 (231.6–792)
Regulatory T cells	37.1 (6.8–88.2)	34.5 (8.7–57.2)
DNT cells	30.8 (0.14–262.5)	39.2 (20.8–80.6)
DPT cells	13.8 (0.08–56.7)	11.9 (3.2–97.3)
NKT cells	128.4 (3.2–453.8)	99.2 (47.9–564.6)

RA, RapidArc irradiation technique; WBC, white blood cell; ALC, absolute lymphocyte count; NK, natural killer; DNT, double-negative T; DPT, double-positive T.

### Statistical Analysis

We evaluated, at every time point, the possible relationship between the clinical covariates and the variation of lymphocyte subpopulations by the linear regression model. We applied a false discovery rate (FDR) approach and the Benjamini–Hochberg method for multiple comparisons correction.

We have checked the normal distribution of immune cell subpopulation values by Shapiro–Wilk’s normality method. Since not all data had a normal course, we chose to represent them by median and interquartile range (IQR) and to apply the non-parametric statistical Kruskal–Wallis test and Wilcoxon test. Statistical significance was considered on a 0.05 cutoff. All the statistical analyses were performed by R software and CRAN packages (33); customized images were processed by the ggpubr package (34).

## Results

**Figure 1:**
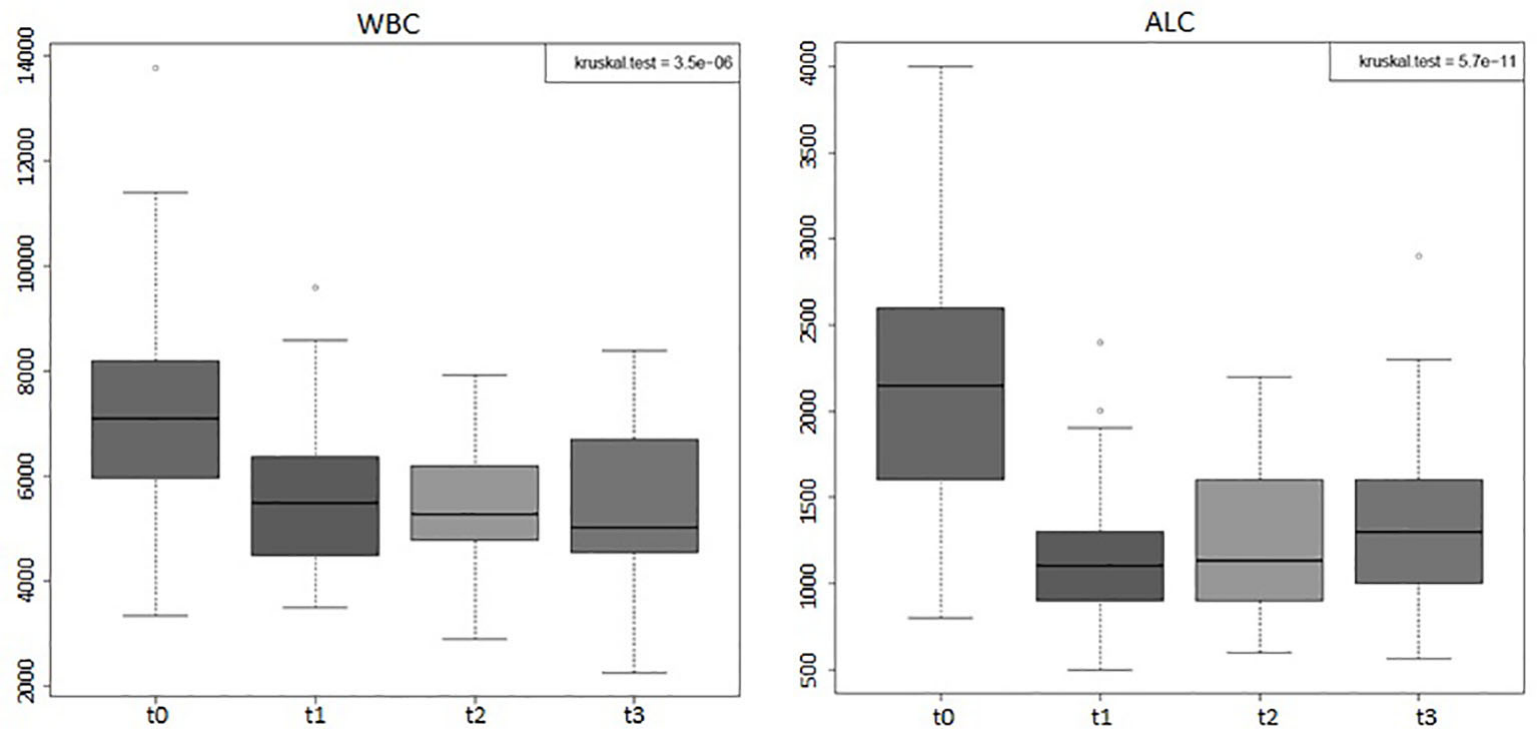
Boxplots of the absolute white blood counts (WBCs) and absolute lymphocyte counts (ALCs) of the entire group of prostate cancer patients (44) who underwent curative RT. The data shown evidenced variations statistically significant during the four time points considered (Kruskal–Wallis test, *p*-value < 0.001). t0: before RT; t1: end of RT; t2: 3 months after RT; t3: 6 months after RT. All values are expressed as cells/µl.

**Figure 2:**
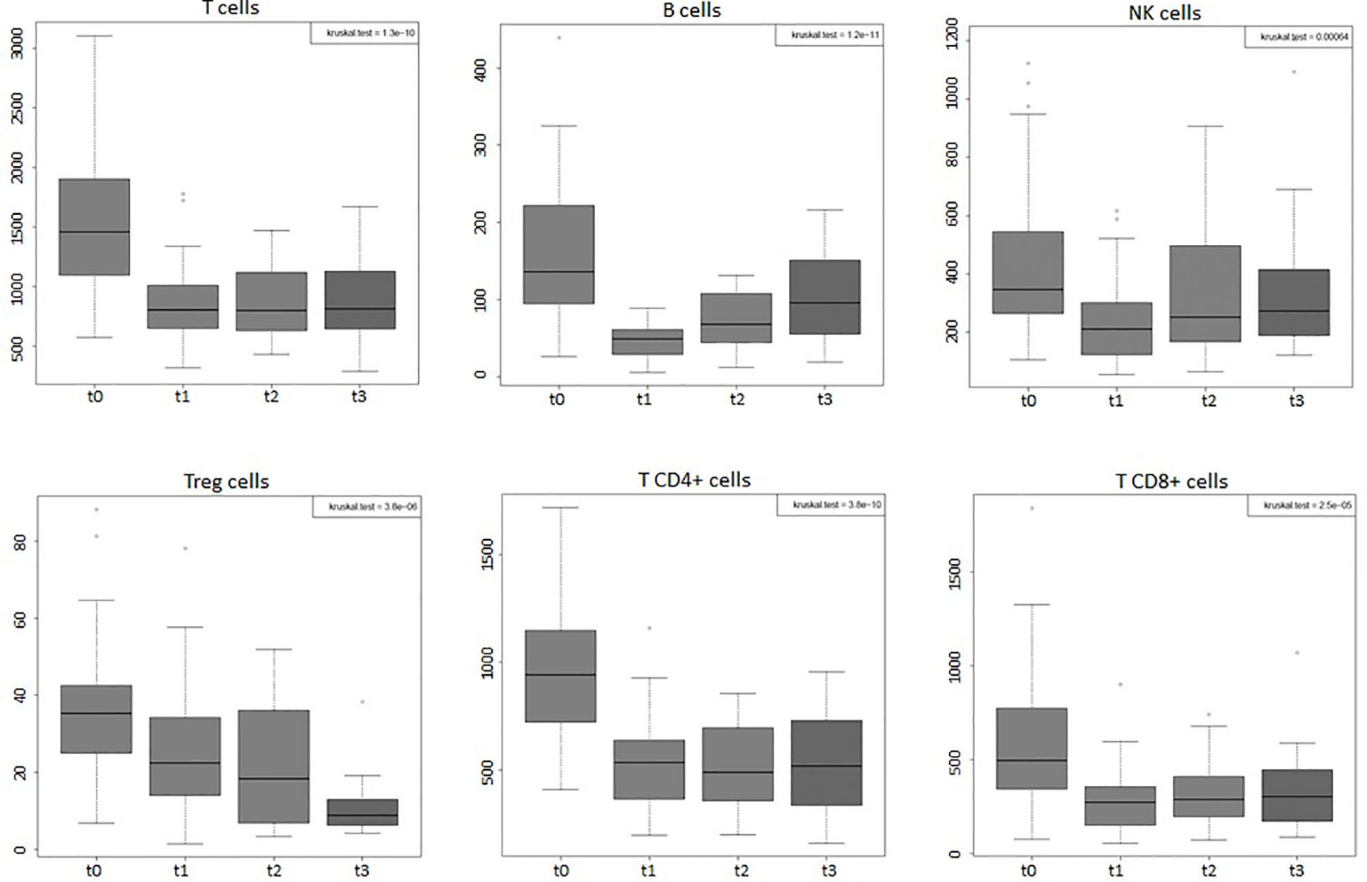
Boxplots of the absolute values of total T, B, NK, Tregs, T CD4, and T CD8 cells of the entire group of prostate cancer patients (44) undergoing curative RT. The data shown evidenced variations statistically significant during the four time points considered (Kruskal–Wallis test, *p*-value < 0.001). t0: before RT; t1: end of RT; t2: 3 months after RT; t3: 6 months after RT. All values are expressed as cells/µl.

**Figure 3:**
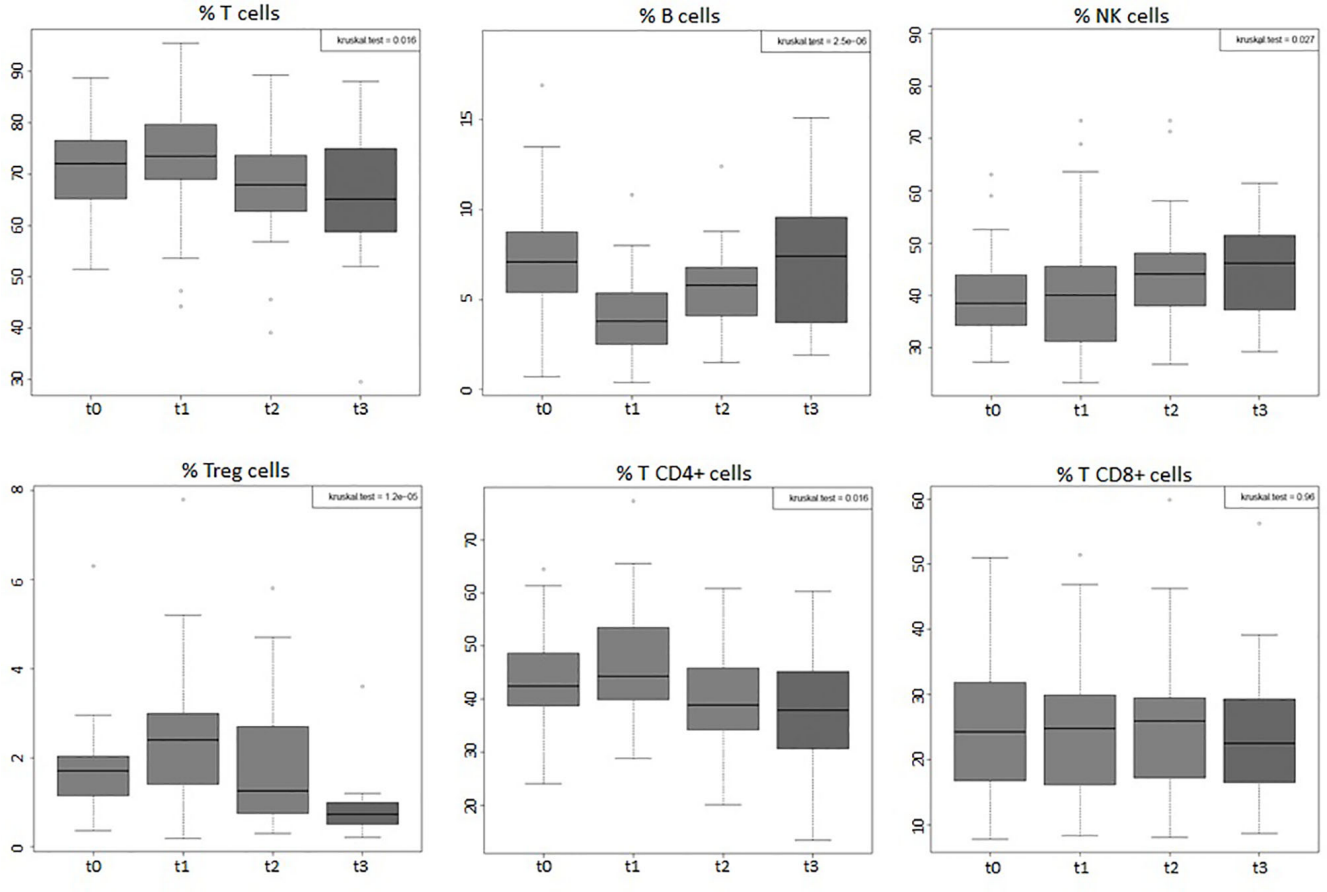
Boxplots of the percentages, among total lymphocytes, of total T, B, NK, Tregs, T CD4, and T CD8 cells of the entire group of prostate cancer patients (44) undergoing curative RT. The data shown evidenced variations statistically significant during the four time points considered (Kruskal–Wallis test, *p*-value < 0.05), except for T CD8+ cells that did not undergo significant changes. t0: before RT; t1: end of RT; t2: 3 months after RT; t3: 6 months after RT.

**Figure 4:**
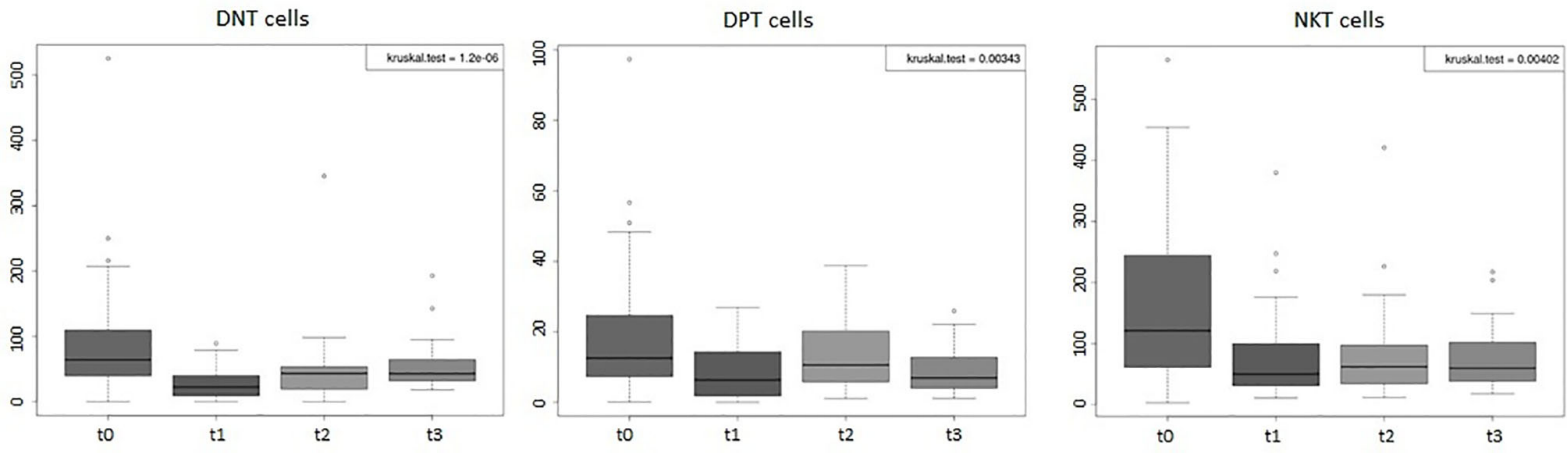
Boxplots of the absolute values of double-negative T (DNT), double-positive T (DPT), and NKT cells of the entire group of prostate cancer patients (44) undergoing curative RT. The data shown evidenced variations statistically significant during the four time points considered (Kruskal–Wallis test, *p*-value < 0.005). t0: before RT; t1: end of RT; t2: 3 months after RT; t3: 6 months after RT. All values are expressed as cells/µl.

Considering the entire group of patients (44), the absolute values of WBC, of lymphocytes, and of all the peripheral immune cell subpopulations (total T, B, NK, Tregs, T CD4+, and T CD8+) displayed significant variations (*p*-value < 0.001) during the 4 time points considered (**Figures 1**, **2**). Percentages of total T, B, NK, Tregs, and T CD4 cells significantly changed (*p*-value < 0.05) during the four time points considered, too (**Figure 3**). RT significantly affected the absolute values of the three small T-cell populations of DNT, DPT, and NKT, as shown in **Figure 4** (*p*-value < 0.05).

When comparing the two groups of patients (hypoF and standard), at 6 months’ follow-up time (t3), the hypoF group showed significantly higher absolute values of total T (linear regression model, adjusted *p*-value: 0.03) and T CD4+ cells (adjusted *p*-value: 0.005) than the standard group. We observed this difference between the two groups also for the absolute values of B cells (adjusted *p*-value: 0.014) but, in particular, at the end of RT (t1). Treg values were also higher in the hypoF group with respect to the standard one at the t2 time point (adjusted *p*-value: 0.03). No statistically significant differences, in terms of WBC, ALC, or peripheral immune cell subpopulations, were found when comparing patients who received hormone therapy with those who did not (data not shown).

**Figure 5:**
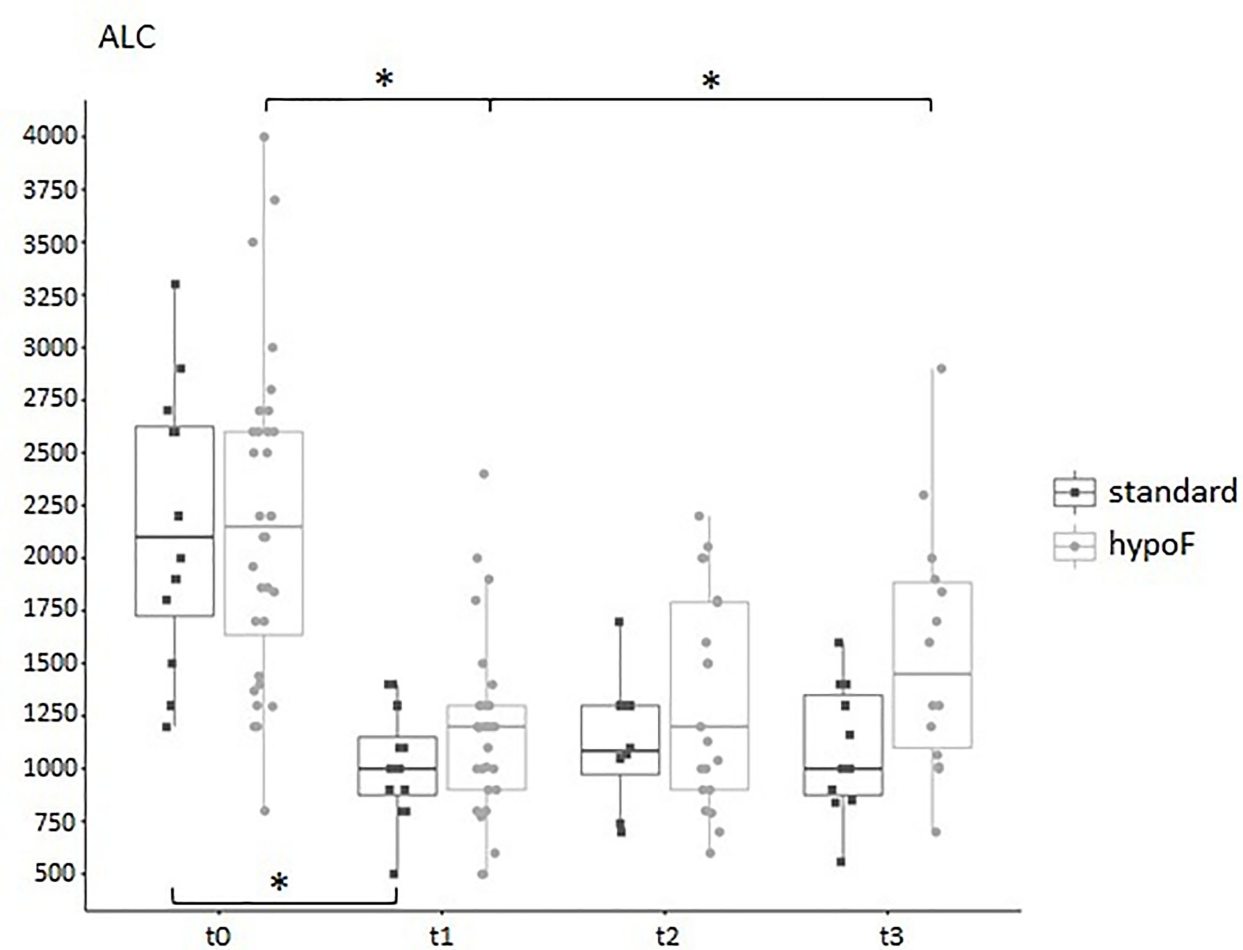
Boxplot of the absolute lymphocyte counts, expressed as cells/µl, in standard (*n* = 12) and hypoF (*n* = 32) RT groups of patients during the four time points considered. Statistically significant differences were marked (Wilcoxon test, *adjusted *p*-value < 0.01).

**Figure 6:**
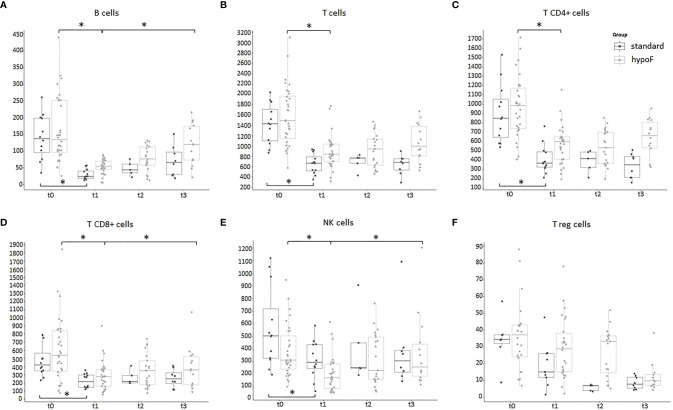
Boxplots of the peripheral immune cell subpopulation values, expressed as cells/µl, in standard (*n* = 12) and hypoF (*n* = 32) RT groups during the four time points considered. **(A)** CD19+ B cells; **(B)** CD3+CD16-CD56- T cells; **(C)** CD4+CD3+ T helper cells; **(D)** CD8+CD3+ T cytotoxic cells; **(E)** CD3-CD16+CD56+ NK cells; **(F)** CD4+CD25+CD127- T reg cells. Statistically significant differences were marked (Wilcoxon test, *adjusted *p*-value < 0.01).

As shown in **Figure 5**, ALC dropped significantly in both groups at the end of RT (t0 vs. t1, standard: adjusted *p-*value: 0.0042; hypoF: adjusted *p-*value: 5.18e-06). At t3, ALC (median: 1,450/µl, IQR: 1,885–1,099/µl) partially recovered with respect to t1 (median: 1,200/µl, IQR: 1,300–900/µl) in the hypoF group (adjusted *p-*value: 0.0094), but not in the standard one in which we did not observe any statistically significant difference at t1 (median: 1000/µl, IQR: 1,150–875/µl) with respect to t3 (median: 1,000/µl, IQR: 1,350–875/µl) (adjusted *p-*value: 0.418). For B cells also, as depicted in **Figure 6A**, there was a significant decrease at the end of RT (t1) with respect to the basal values (t0), in both groups (t0 vs. t1, standard: adjusted *p-*value: 0.00097; hypoF: adjusted *p-*value: 1.49e-07). This subpopulation, in the hypoF group, at t3 was 88.8% (median: 120/µl, IQR: 174–73/µl) of the t0 value (median: 135/µl, IQR: 251–97/µl). In the standard group, instead, at t3, the B-cell absolute value was still 48.2% (median: 66/µl, IQR: 97–30/µl) of the t0 value (median: 137/µl, IQR: 198–95/µl). Total T cells (t0 vs. t1, standard: adjusted *p-*value: 0.00097; hypoF: adjusted *p-*value: 1.49e-07) and subpopulations of T CD4+ (t0 vs. t1, standard: adjusted *p-*value: 0.00097; hypoF: adjusted *p-*value: 1.88e-07) and T CD8+ (t0 vs. t1, standard: adjusted *p-*value: 0.0015; hypoF: adjusted *p-*value: 5.21e-07) also decreased significantly at t1 with respect to t0 in both groups (**Figures 6B–D**). The values of these three peripheral immune cell populations partially recovered at t3 with respect to t1 only in the hypoF group (statistically significant only for T CD8+ cells, adjusted *p-*value: 0.0091) (**Figures 6B–D**). As regards the NK lymphocyte population, there was a great difference in values, but not statistically significant, before the beginning of RT between the two groups of patients, not detected for ALC or for the other immune cell subpopulations. At the end of RT, the NK cell absolute values significantly dropped in both groups of patients (t0 vs. t1, standard: adjusted *p*-value: 0.0055; hypoF: adjusted *p-*value: 5.24e-07), and during follow-up, such values partially recovered only in the hypoF group (**Figure 6E**). We found no significant difference in Treg lymphocyte absolute values between the two groups (data not available for all patients and all time points) during the 4 time points considered. As shown in **Figure 6F**, their values were lower at 6 months’ follow-up (t3) than at the end of RT (t1).

**Figure 7:**
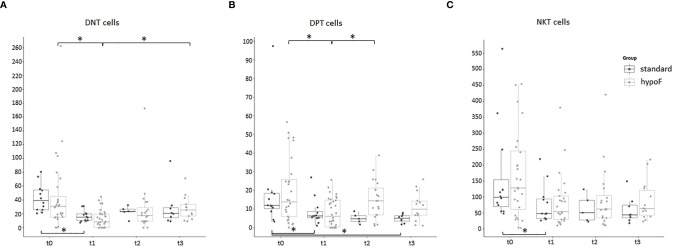
Boxplots of the 3 small T-cell population values, expressed as cells/µl, in standard (*n* = 12) and hypoF (*n* = 32) RT groups during the four time points considered. **(A)** CD3+CD4-CD8-CD16-CD56- double-negative T cells (DNT); **(B)** CD3+CD8+CD4+ double-positive T cells (DPT); **(C)** CD3+CD16+CD56+ NKT cells. Statistically significant differences were marked (Wilcoxon test, *adjusted *p*-value < 0.05).

As regards DNT cells (**Figure 7A**), in the hypoF group, we found that their values decreased significantly following RT (t1) (t0 vs. t1, adjusted *p-*value: 0.00079). During follow-up time points, we observed a statistically significant increase in values (t1 vs. t3, adjusted *p-*value: 0.0073). We observed the same trend of the DNT values (**Figure 7A**) also in the standard group at the end of RT (t0 vs. t1, adjusted *p-*value: 0.0087), with an increase in values (not statistically significant) during follow-up time. For the immune cell population of DPT (**Figure 7B**), in the hypoF group, we observed a reduction of values at t1 (t0 vs. t1, adjusted *p-*value; 0.028) and a recovery of values (t1 vs. t2, adjusted *p-*value: 0.037). In the standard group, DPT values dropped down significantly at t1 (t0 vs. t1, adjusted *p-*value: 0.038), and they remained almost stable during follow-up (**Figure 7B**). NKT cells decreased in the hypoF group at the end of RT but not significantly, probably due to large variability in baseline values. In the standard group, we registered a statistically significant reduction of NKT values at t1 with respect to t0 (adjusted *p-*value: 0.0058) (**Figure 7C**).

Considering the entire group of patients enrolled, the median follow-up time was 18 months (range, 6–36 months), in particular 28 months (range, 6–36 months) for the standard group and 18 months (range, 6–36 months) for the hypoF group. Biochemical free-survival (BFS) was 97.7% (100% for the standard group and 96.8% for the hypoF group) as only one patient, in the hypoF group, relapsed 6 months after the end of RT.

As regards genitourinary (GU) and gastrointestinal (GI) toxicity (35), we did not find any significant difference between the two techniques/fractionation regimens, as also none of the immune cell subpopulations, ALC, or WBC values correlated to acute or late GU or GI toxicity. We registered, instead, a significantly higher number of late Grade 1 leukopenias (35) at t3 time points in the standard group with respect to the hypoF group (81.8% vs. 21.4%, linear regression model, adjusted *p*-value: 0.0065). All toxicity data are reported in **Table 3**.

**Table 3:** Toxicity data.

	RA/hypofractionation RT group (*n* = 32)		3D conformal/conventional fractionation RT group (*n* = 12)	
	Grade 1	Grade 2	Grade 1	Grade 2
Acute toxicity				
Genitourinary	9 (20.4%)	2 (4.5%)	6 (13.6%)	2 (4.5%)
Gastrointestinal	11 (25%)	3 (6.8%)	6 (13.6%)	3 (6.8%)
Hematological (lymphopenia)	4 (9%)	4 (9%)	4 (9%)	1 (2.3%)
Late toxicity				
Genitourinary	9 (20.4%)	0	1 (2.3%)	0
Gastrointestinal	3 (6.8%)	1 (2.3%)	1 (2.3%)	1 (2.3%)
Hematological (lymphopenia)	0	1 (2.3%)	3 (6.8%)	1 (2.3%)

RA, RapidArc irradiation technique; RT, radiotherapy.

## Discussion

Leukopenia and lymphopenia are two side effects of WPRT, well known by radiation oncologists and well documented in literature (15, 17, 36, 37). The purpose of our study was to compare the modulation of peripheral immune cell subpopulation values by two different RT technique/fractionated regimens commonly used to treat localized prostate cancer patients. We found that WBCs, and particularly ALCs, are significantly affected by RT and greatly decreased at the end of the treatment. Sanguineti et al. (38) have recently reported a WBC count depression in prostate cancer patients following RT treatment in moderate hypofractionation with respect to conventional fractionation. In our study, ALC values showed a trend to recover 6 months after the end of RT in the hypoF group but not in the standard group, in which the number of Grade 1 leukopenias was significantly higher at this time point. Hematologic toxicity, in particular lymphopenia, in this setting of patients was also found to be prolonged and not negligible by Cozzarini et al. (37), who, anyway, did not find differences among 3 different intensity modulated radiation therapy (IMRT) modalities (Helical Tomotherapy, RA, Static Field-IMRT). The same authors showed that a higher risk of acute or late lymphopenia was associated with higher bone marrow volumes receiving ≥40 Gy (V40) (15). With regard to the different immune cell subpopulations, we found less toxicity of the RA/hypofractionation technique for B cells at t1 and for total T and T CD4+ at t3 with respect to conformal/conventional fractionation RT. The B-cell population was the only one that decreased in both percentage (**Figure 3**) and absolute (**Figure 2**) values after RT and appeared to be mostly affected at the end of RT with both technique/fractionation regimens (**Figure 6A**). This finding is in line with previous studies that documented the effects of RT on lymphocyte subpopulation composition, and in particular on B cells, in breast cancer patients (18, 25) and in patients undergoing pelvis RT (17, 19, 20), particularly for prostate cancer (21–24). Since they mature in the bone marrow (39), in fact, B cells are the most vulnerable to RT directed towards the pelvis bones. Moreover, we found that B cells display the highest rate of recovery 6 months after the end of RT (t3) (89% and 48% of the baseline values in the hypoF and standard group, respectively) with respect to the other lymphocyte subpopulations. Three and six months after the end of RT, total T, T CD4+, T CD8+, and NK lymphocytes, in fact, tended to have almost the same absolute values observed at the end of RT in the standard group and showed a limited recovery in the hypoF group. The only exception was the Treg population that displayed lower absolute values at the follow-up time of 6 months with respect to the end of RT in both groups. A previous study (23) conducted on 18 PC patients who underwent normofractionated RT reported that the percentage of total T cells, T CD8+, and T CD4+ decreased during RT, while Treg and NK cells increased. The proportion of NK and total T cells remained significantly altered at 3 months’ follow-up. They did not investigate the absolute values of the single immune population in the peripheral blood.

Changes in immune cell subpopulations, in particular T lymphocytes, following RT is currently a topic of interest. In this context, a recent meta-analysis by Wang et al. summarized the effects of RT in different types of tumor, concluding that during the first month of RT, the values of T lymphocytes undergo an evident reduction due to apoptosis (40).

Our study is the first to perform a longitudinal monitoring of the effects of RT on small populations of T lymphocytes DNT, DPT, and NKT.

These populations have already been previously studied in the cancer setting. Two different groups reported a significant decrease in peripheral DNT cells, respectively, in metastatic melanoma and multiple myeloma patients compared to healthy controls (41, 42). DPT cells with cytotoxic potential were found in great numbers in pleural effusion in human breast cancer patients and were described to play a suppressive role in colorectal cancer (31). NKT-like cell values were also reduced in colorectal cancer (27). Regarding DNT, in both groups, we found a significant decrease in values at the end of RT treatment. DNT values then tend to increase during 6 months’ follow-up. The DPT cells also dropped down significantly in both groups at the t1 time point, but then remained almost stable during follow-up in the standard group while they have an increase in the hypoF group. The NKT cell values, after the decrease observed at the end of RT, remained stable up to 6 months after the end of RT.

To the best of our knowledge, this is the first study to discuss the effects of RapidArc/moderate hypofractionation curative RT on immune cell subsets in patients treated for localized prostate cancer. Furthermore, we also tried to compare these effects with those due to 3D conformal/conventional fractionation RT, albeit in a limited number of patients.

In the future, we intend to also elucidate the possible correlation between changes in immune cell populations and irradiated volumes as well as other RT parameters.

In conclusion, our preliminary findings, obtained in a small subset of prostate cancer patients, suggest a lower hematologic and immunologic toxicity of the RA/hypofractionation technique with respect to conformal/conventional fractionation RT in terms of ALC as well as of the different peripheral immune cell subpopulations, which play several roles in innate and adaptive immunity. In the era of personalized medicine, it is challenging to study the different RT effects on immune system cells, particularly in those clinical settings in which there is a possibility to combine RT with immunotherapy.

## Data Availability Statement

The datasets presented in this article are not readily available because of the participants’ identifiable data. Requests to access the datasets should be directed to fiore.dauria@virgilio.it.

## Ethics Statement

The studies involving human participants were reviewed and approved by Comitato Etico Unico Regionale per la Basilicata. The patients/participants provided their written informed consent to participate in this study.

## Author Contributions

Design of the work: LR and VF. Statistical analysis: GCal. Investigation and interpretation of data: FD’A, TS, and LV. Acquisition of data: GB and AT. Acquisition of data and organization of the database: AM, GCas, IS, AZ, and TV. Writing—original draft: FD’A and LR. Writing—review and editing: AS and GCal. All authors contributed to the article and approved the submitted version.

## Conflict of Interest

The authors declare that the research was conducted in the absence of any commercial or financial relationships that could be construed as a potential conflict of interest.

## Publisher’s Note

All claims expressed in this article are solely those of the authors and do not necessarily represent those of their affiliated organizations, or those of the publisher, the editors and the reviewers. Any product that may be evaluated in this article, or claim that may be made by its manufacturer, is not guaranteed or endorsed by the publisher.
